# Healthy mental higher education students’: Presentation of a project

**DOI:** 10.1192/j.eurpsy.2023.2095

**Published:** 2023-07-19

**Authors:** A. Torres, J. Costa, P. Carvalho, M. Loureiro

**Affiliations:** 1 Department of Psychology and Education, University of Beira Interior, Covilhã; 2 Department of Education and Psychology, Center for Health Technology and Services Research (CINTESIS@RISE), Aveiro, Portugal

## Abstract

**Introduction:**

The prevalence of mental disorders in Higher Education Students (HES) is high and has shown a worrying growth. However, only a small percentage of HES in need of psychological support request it, due to the stigma related to mental illness, requesting informal help from friends and family. Training and increasing Mental Health Literacy (MHL) have been stated as appropriate strategies to reduce stigma and increase the demand for professional psychological support. However, with the increase in the demand for help, the difficulty of providing an adequate response from psychological support of HE services also increases. Therefore, procedures that develop socio-emotional skills in the HES and strategies that enhance the responsiveness of these services are necessary.

**Objectives:**

Taking in consideration the literature review in this field, we develop a project to improve the mental health of HES. The project objectives’: a) the reduction of stigma related to mental disorders through the promotion of MHL in the academic community; b) the promotion of mental health and socio-emotional skills of HES; c) increasing detection and active search for professional mental health support, and d) the implementation of psychological intervention based on a stepped care model that provides an adequate response to most of the students’ mental health needs, according to individual needs.

**Methods:**

The project proposes to perform the following methods: a) training in mental health open to the academic community, which will aim to train volunteers to be Gatekeepers, in order to promote awareness, detection and referral of students in need of professional psychological support; b) implementation of a Student Observatory, with tracking of psychopathological symptoms, supported on the Web; c) implementation of a psychological intervention program based on a stepped care model, which will include the following progressive phases by severity: 1) digital self-help manual; 2) web-based self-help groups; 3) psychological intervention groups supported by the Web; 4) face-to-face intervention groups and 5) individual psychological intervention.

Students will be assessed before and after each step of care, with the following psychological instruments: Mental Health Inventory (MHI); Patient Health Questionnaire (PHQ-9) and Generalized Anxiety Disorder (GAD-7).

**Results:**

The implementation of the presented methods expect to achieve improvements on HES’ mental health, namely improvement of MHI, reduction of PHQ-9 and GAD-7 after each step of the psychological care.

**Conclusions:**

The project presented encloses evidence-based interventions, with inspiration on psychoeducation and cognitive behavioral approaches, and it is expected to contribute to the improvement of mental health of HES. The results will be collected and disseminated. We encourage other researcher and clinicians to perform studies about the mental health of HES.

**Disclosure of Interest:**

None Declared

